# Organocatalysis in Synthesis: L-Proline as an Enantioselective Catalyst in the Synthesis of Pyrans and Thiopyrans

**DOI:** 10.3390/molecules17044300

**Published:** 2012-04-10

**Authors:** Noha M. Hilmy Elnagdi, Noura Saad Al-Hokbany

**Affiliations:** Women Students-Medical Studies & Sciences Sections, Chemistry Department, College of Science, King Saud University, Riyadh, KSA, P.O. Box 22452, Riyadh 11495, Saudi Arabia

**Keywords:** L-proline, pyran, assymetric synthesis, enantioselectivity, ethyl propiolate, optical activity

## Abstract

The multicomponent reaction (MCR) of aromatic aldehydes **1** and malononitrile (**2**) with active methylenes **5a–h** in the presence of L-proline produced pyrans and thiopyrans **6a–h** stereospecifically and in good yields. Moreover a novel MCR of ethyl propiolate (**8**) with **1** and **2** in the presence of L-proline to afford (*R*)-polysubstituted pyran is also reported. X-ray structures, e.e. and optical activity of the synthesized compounds indicated that L-proline as a catalyst is responsible for the observed enantioselectivity in the studied reactions.

## 1. Introduction

Polyfunctionally substituted pyrans are no doubt an important class of heterocycles due to their great biological and pharmacological importance [1,2,3,4,5,6]. The addition of active methylene reagents to arylidenemalononitrile in the presence of homogeneous basic catalysts has been extensively used in the past for the synthesis of these compounds [7,8,9,10,11,12]. Interest in these reactions has recently been revived [13,14] with the aim of developing green laboratory reaction conditions [15,16], such as replacing homogeneous catalysis with heterogeneous ones [17,18,19,20], to synthesize enantiomerically pure pyrans for which diverse biological applications were noticed [21,22,23] and patented [24,25,26]. Many of these new approaches use multicomponent reactions and either an organocatalyst [27,28] or sometimes metal or nanoparticulated catalysts [29,30,31]. Although in plenty of these reactions a chiral center is being created only a few published works have discussed the exact stereochemistry of the synthesized compounds. 

Since L-proline is a readily obtainable naturally occurring amino acid and is easy to obtain in high enantiomeric purity it has been reported as an eco-friendly catalyst for the synthesis of several heterocycles [32,33,34,35,36,37]. Recently Muramulla *et al.* reported the use of modularly designed organocatalysts (MDO) of L-proline in dichloromethane as a solvent for the synthesis of chiral pyranopyrazoles in moderate e.e. [38]. Gou *et al*. have also reacted aromatic aldehydes, malononitrile, and dimedone, in the presence of D,L-proline as a catalyst in the absence of solvent to obtain 2-amino-3-cyano-4-aryl-7,7-dimethyl-5,6,7,8-tetrahydrobenzo[b]pyran [27].

It seemed thus of value to see if the use of L-proline as a catalyst in the reaction of active methylene ketones with α,β-unsaturated nitriles in MCRs can be used to induce enantioselectivity of the synthesized pyrans. In this article the syntheses of pyrans, condensed pyrans and thiopyrans are reported. Moreover a novel pyran was prepared via the MCR of ethyl propiolate (**8**) with aldehydes and malononitrile in the presence of L-proline as a catalyst.

## 2. Results and Discussion

**Scheme 1:**
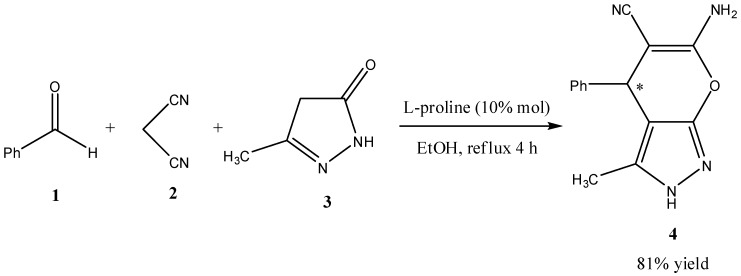
Synthesis of 6-amino-3,4-dimethyl-4-phenyl-2,4-dihydropyrano[2,3-c]pyrazole-5-carbonitrile (**4**).

First in an attempt to synthesize the chiral pyranopyrazoles **4**, we have reacted benzaldehyde (**1**), malononitrile (**2**) and pyrazolon-5-one (**3**) with 10% mol L-proline as the only catalyst. In contrast to Muramulla *et al*’s. findings that for the same reaction using L-proline alone as a catalyst under the reported reaction conditions no product was obtained, in our case after the reaction mixture was refluxed in ethanol for 4 h, the pyranopyrazole **4** was isolated in 81% yield (Scheme 1). To initially test if L-proline has induced any enantioselectivity in this reaction, compound **4** was tested for optical activity and found to be optically active with a specific rotation of +247.02 ([α]_D_, 25 °C, *c* = 1, DMF).

**Scheme 2:**
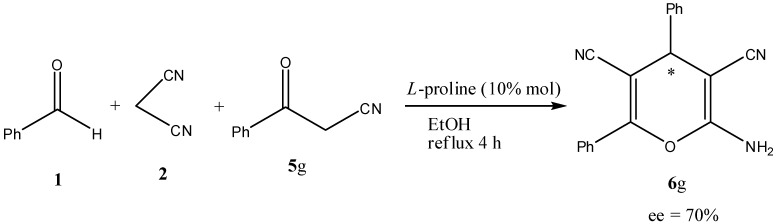
Synthesis of 2-amino-4,6-diphenyl-4*H*-pyran-3,5-dicarbonitrile (**6g**).

Next reacting benzaldehyde (**1**), malononitrile (**2**) and 3-oxo-3-phenylpropanenitrile (**5g**) in the presence of 10% L-proline as a catalyst afforded 2-amino-4,6-diphenyl-4*H*-pyran-3,5-dicarbonitrile (**6g**) in 83% yield and 70% e.e. (Scheme 2).

The 4*H*-pyran **6g** was found to have 70% e.e. Confirmation that the 4*H*-pyran **6g** indeed displayed an enantiomeric excess was obtained by performing ^1^H-NMR experiments with **6g** in the presence of a chiral shift reagent (europium tris[3-heptafluoropropylhydroxymethylene]-(‏+)-camphorate). After making successive additions of this chiral shift reagent to a CDCl_3_ solution of **6g**, the 4*H-*proton at δ_H_ 4.7 ppm appeared to resolve into two components (most obviously after the addition of 6 mg of the chiral shift reagent), and by calculation of the area under the chosen peak from the ^1^H-NMR that showed the maximum separation of the two components, **6g** was found to be in 70% e.e. However at this stage, we cannot judge the predominance of the *R* or *S* enantiomers for this compound.

**Scheme 3:**
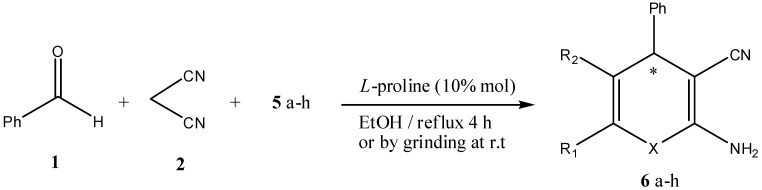
Synthesis of enantioselective pyrans, benzopyrans, and thiopyrans **6a–h** in a multicomponent reaction using L-proline as a catalyst.

The above reported results encouraged us to prepare a series of polysubstituted 4*H*-pyrans. Pyrans **6a–h** were all synthesized by the addition of benzaldehyde (**1**) and malononitrile (**2**) to active methylenes **5a–h** using L-proline (10% mol) as a catalyst in a MCR to afford chiral pyrans **6a–h** (Scheme 3). Active methylenes used are listed in Table 1. Structures and yields of the products **6a–h**, as well as the reaction conditions are listed in Table 2.

**Table 1:** Compounds **5a–h**.

**5a**	CH_3_COCH_2_COOEt	**5e**	EtCOOCH_2_COPh
**5b**	CH_3_COCH_2_COCH_3_	**5f**	PhCH_2_COOCH_2_COCH_3_
**5c**	NCCH_2_CSNH_2_	**5g**	PhCOCH_2_CN
**5d**	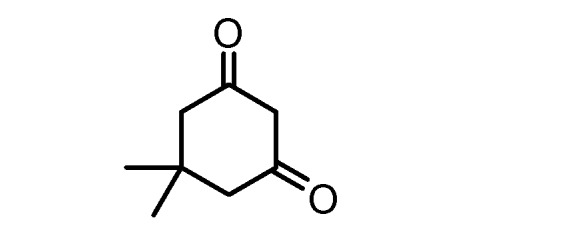	**5h**	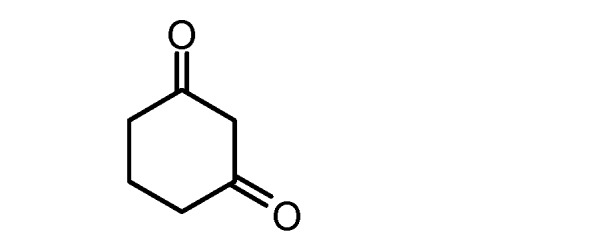

**Table 2:** Compounds **6a–h** and their yields.

Compound ^a^	X	R_1_	R_2_	Yield (%)
**6a**	O	CH_3_	COOEt	72
**6b**	O	CH_3_	COCH_3_	60
**6c**	S	NH_2_	CN	92
6d *	O	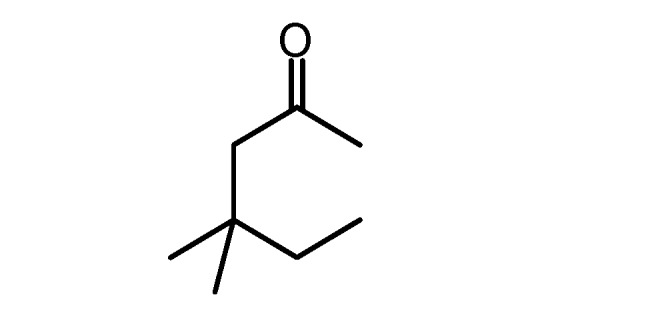		90
**6e**	O	Ph	COOEt	87
**6f**	O	CH_3_	COOCH_2_Ph	65
**6g**	O	Ph	CN	83
6h *	O	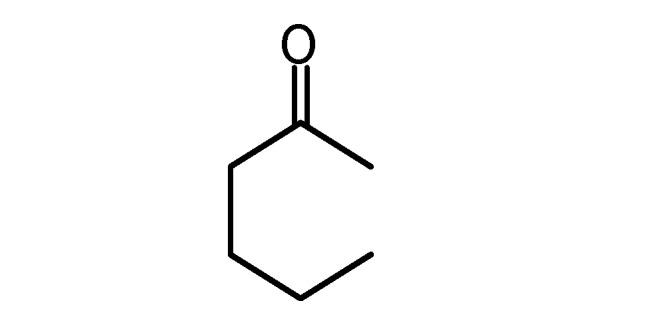		78

^a^ Compounds were characterized by their spectral data (IR, ^13^C-NMR, ^1^H-NMR). * These compounds could be prepared without a solvent at r.t. using grinding for 5 min. Compounds **6a**, **b**, **c**, **e**, **f**, **g** were prepared using EtOH as a solvent and refluxing for 4 h.

Specific rotation measurements for some selected synthesized compounds revealed that these compounds are optically active, which supports the assumption that L-proline when used as a catalyst brings about enantioselectivity in such reactions. The specific rotation of some selected compounds is listed in Table 3.

**Table 3:** Specific rotation for some of the synthesized compounds.

Entry	Specific rotation [α]_D_ 25 °C, *c* = 1, DMF
**6a**	+318.20
**6e**	+198.81
**6h**	+198.20
**4**	+247.02
**13**	+272.0

**Figure 1:**
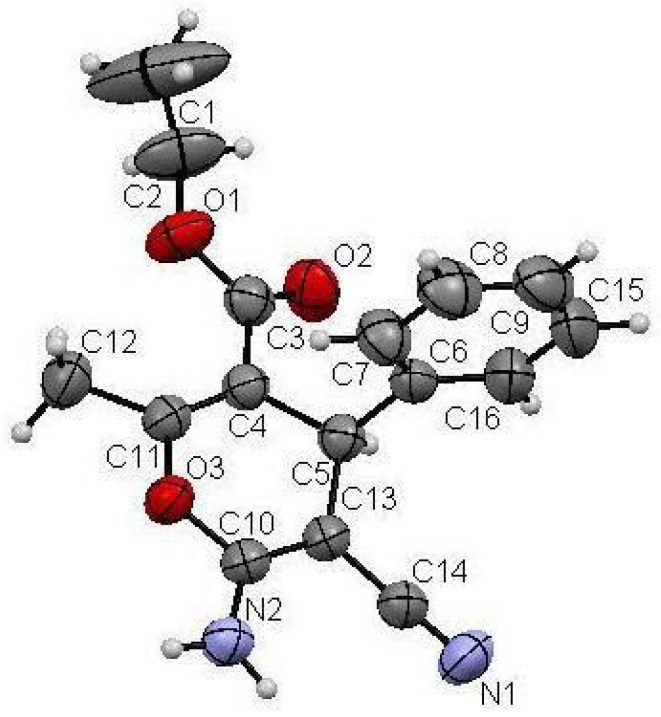
X-ray crystal structure of (*S*)-ethyl 5-cyano-2,6-dimethyl-4-phenyl-4*H*-pyran-3-carboxylate (**6a**).

**Figure 2:**
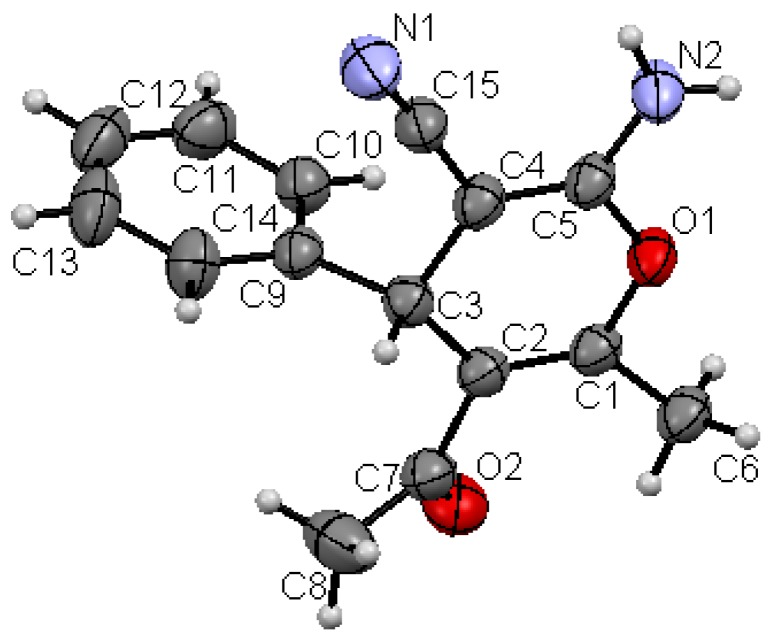
X-ray crystal structure of (*R*)-5-acetyl-2-amino-6-methyl-4-phenyl-4*H*-pyran-3-carbonitrile (**6b**).

**Figure 3:**
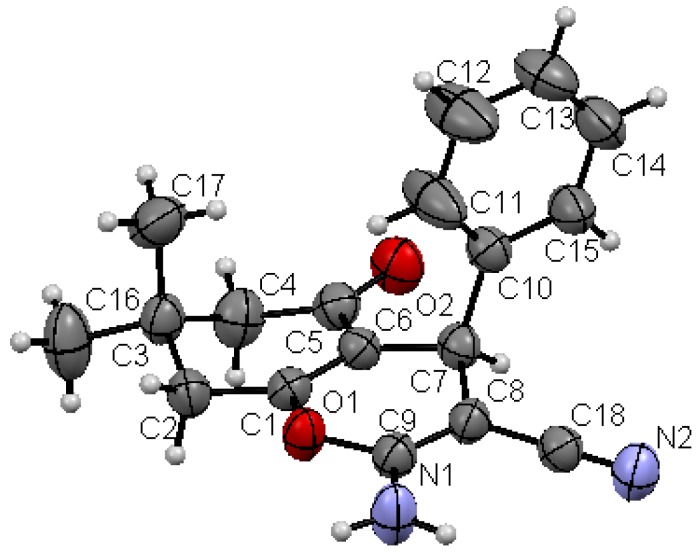
X-ray crystal structure of (*S*)-2-amino-7,7-dimethyl-5-oxo-4-phenyl-5,6,7,8-tetrahydro-4*H*-chromene-3-carbonitrile(**6d**).

**Figure 4:**
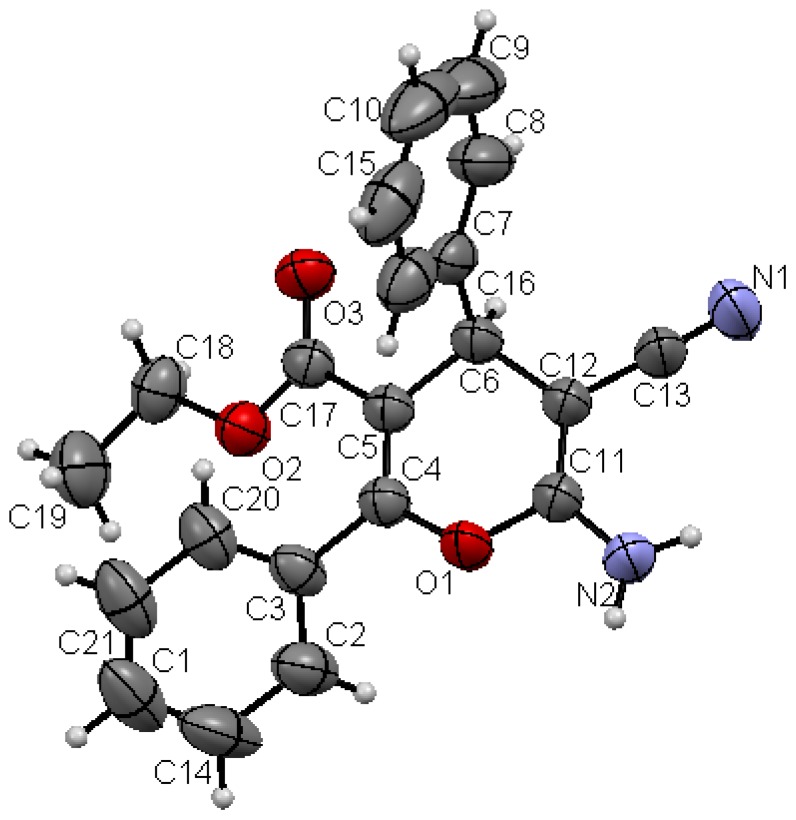
X-ray crystal structure of (*S*)-ethyl 6-amino-5-cyano-2,4-diphenyl-4*H*-pyran-3-carboxylate (**6e**).

**Figure 5:**
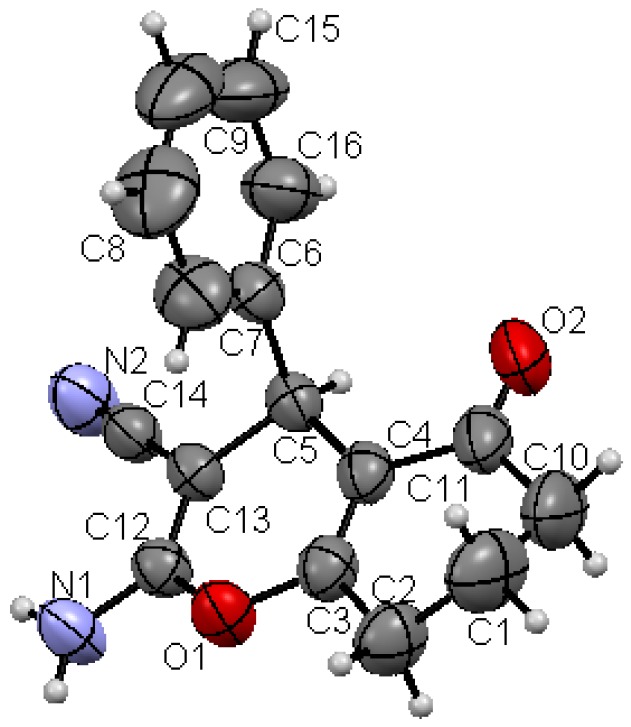
X-ray crystal structure of (*R*)-2-amino-5-oxo-4-phenyl-5,6,7,8-tetrahydro-4*H*-chromene-3-carbonitrile (**6h**).

**Figure 6:**
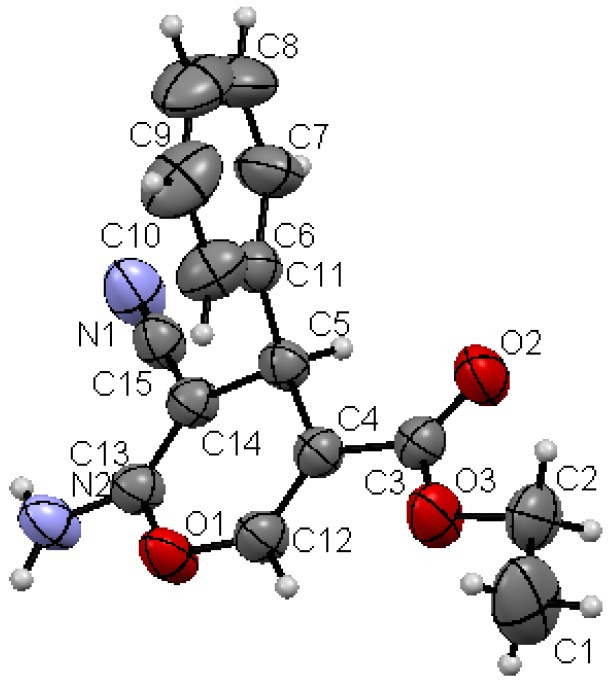
X-ray crystal structure of (*R*)-ethyl-6-amino-5-cyano-4-phenyl-4*H*-pyran-3-carboxylate **13**.

**Scheme 4:**
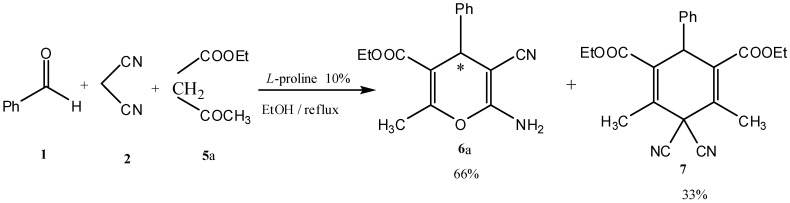
Synthesis of the two products **6a** and **7** in a 2:1 ratio.

Structures proposed of the products **6a**, **b**, **d**, **e**, **h** were well documented by X-ray crystallography as shown in Figure 1, Figure 2, Figure 3, Figure 4, Figure 5, Figure 6 [39]. It is worth mentioning that the ^1^H-NMR of the compound **6a** has revealed the formation of two products in 2:1 ratio that could be separated by column chromatography. The first product could be shown by X-ray crystal structure (Figure 1) to be the 4*H*-pyran derivative (*S*)-ethyl 6-amino-5-cyano-2-methyl-4-phenyl-4*H*-pyran-3-carboxylate (**6a**), while the other product with molecular formula C_22_H_22_N_2_O_4_ and *m/z* = 378.2 is believed to be diethyl 5,5-dicyano-4,6-dimethyl-2-phenylcyclohexa-3,6-diene-1,3-dicarboxylate (**7**) as was proven by its spectroscopic data (Scheme 4).

**Scheme 5:**
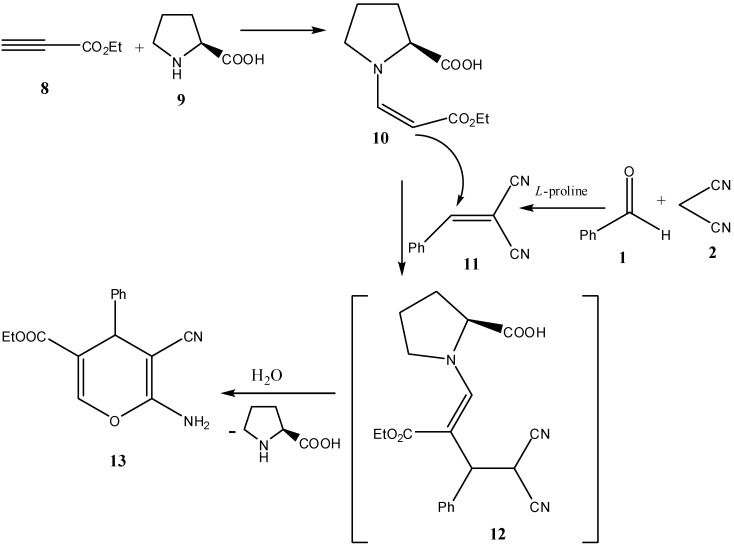
Synthesis of (*R*)-ethyl-6-amino-5-cyano-4-phenyl-4*H*-pyran-3-carboxylate (**13**).

In addition a novel synthesis of pyran **13** could be achieved by mixing benzaldehyde (**1**), and malononitrile (**2**) with ethyl propiolate (**8**) in ethanol and 10% L-proline as a catalyst. It is believed that initially L-proline (**9**) adds to ethyl propiolate (**8**) affording the enamine ester **10**, while benzaldehyde (**1**) condenses with malononitrile (**2**) affording 2-benzylidenemalononitrile (**11**). This was followed by the addition of the electron rich β-carbon in the enamine ester to the electron poor π system in the benzylidine-malononitrile **11**, affording an adduct. This adduct **12** is then hydrolyzed by H_2_O and cyclizes into **13** (Scheme 5). Compound **13** was also tested for optical activity and found to be optically active with a specific rotation of +272.0 ([α]_D_ 25 °C, *c* = 1, DMF). The structure of **13** has been confirmed with certainty via X-ray crystal structure determination (Figure 6).

As shown in the X-ray structures (Figure 1, Figure 2, Figure 3, Figure 4, Figure 5, Figure 6), we have obtained the *R*-enantiomer in the case of compounds **9a**, **h**, and **14**, and the *S*-enantiomer in the cases of **9a**, **d**, and **e**, but both enantiomers exist of course in the original product as a mixture.

## 3. Experimental

### 3.1. General

The ^1^H-NMR and ^13^C-NMR spectra were determined by using a Bruker DPX instrument at 400 MHz for ^1^H-NMR and 100 MHz for ^13^C-NMR. The chemical shifts are reported in ppm downfield to TMS (δ = 0) or DMSO-D_6_ (δ = 2.5) for ^1^H-NMR and relative to the central CDCl_3_ resonance (δ = 77.0) or DMSO-D_6_ (δ = 40.0) for ^13^C-NMR. The coupling constants *J* are given in Hz. Mass spectra were measured using a high resolution GC-MS (DFS) Thermo spectrometer with EI (70 EV). Column chromatography was performed using Acme’s silica gel (particle size 0.063–0.200 mm). IR spectra were recorded using KBr disks on a Perkin-Elmer System 2000 FT-IR spectrophotometer. Microanalyses were performed on a LECO CHNS-932 Elemental Analyzer. Optical rotations were measured on an Autopol IV (Rudolph Instruments) automatic polarimeter at 25 °C in DMF at concentration 1 mol. X-ray crystal structures were determined using a Single Crystal X-ray Crystallography-Rigaku Rapid II system and all the X-ray samples were prepared by recrystallization from hot ethanol. All melting points were recorded on a Griffin melting point apparatus and are reported uncorrected.

### 3.2. General Experimental Procedure for the Synthesis of Pyrans **6a–h** and Compound **7**

A mixture of benzaldehyde (**1**, 0.01 mol), malononitrile (**2**, 0.01 mol) and 10% mol L-proline was stirred at r.t. for 2 min. then active methylenes **5a–h** (0.01 mol) were added. The mixture was refluxed in ethanol (10 mL) for 4–6 h followed by TLC. The crude compounds formed were recrystallized from ethanol and further purified using column chromatography using 2:1 petroleum ether/ethyl acetate as an eluent.

*Ethyl-5-cyano-2,6-dimethyL-4-phenyl-4H-pyran-3-carboxylate *(**6a**). White crystalline solid, Mp 189–190 °C; yield 72%, ^1^H-NMR [DMSO-d_6_], δ: ppm = 7.31 (t, 1H, Ar), 7.23 (m, 1H, Ar), 7.15 (d, *J* = 7.6 Hz, 1H, Ar), 6.94 (s, 2H, NH_2_), 4.29 (s, 1H), 3.94 (m, 2H, CH_2_), 2.30 (s, 3H, CH_3_), 1.03 (t, 3H, CH_3_); ^13^C-NMR: δ: ppm = 166 (O=C), 158.8 (C), 156.6 (C), 144.8 (CH), 128.5 (2C), 127.0 (2C), 126.9 (2C), 119.5 (CN), 107.0 (C), 60.0 (CH_2_), 58.8 (C), 19.6 (CH_3_), 17.0 (CH_3_); MS: *m/z* % 284.1 (M+100); Anal. calcd for C_16_H_16_N_2_O_3_ (284.30): C, 67.59; H, 5.67; N, 9.85; O, 16.882. Found: C, 67.63; H, 5.66; N, 9.71%.

*5-Acetyl-2-amino-6-methyl-4-phenyl-4H-pyran-3-carbonitrile *(**6b**). White crystalline solid, Mp 185–186 °C; yield 72%, ^1^H-NMR [DMSO-d_6_] δ 7.34 (m, 2H, Ar), 7.23 (m, 1H, Ar), 7.18 (m, 2H, Ar), 6.87 (s, 2H, NH_2_), 4.46 (s, 1H), 2.25 (s, 3H, CH_3_), 2.06 (s, 3H, CH_3_); ^13^C-NMR: [DMSO-d_6_], δ: ppm = 198 (O=C), 158.2 (C), 154.8 (C), 144.5 (CH), 128.7 (2C), 127.1 (2C), 126.9 (2C), 119.8 (CN), 114.9 (C), 57.7 (C), 29.8 (CH_3_), 18.4 (CH_3_); MS: *m/z* % 254.1 (M+100); Anal. calcd for C_15_H_14_N_2_O_2_ (254.1): C, 70.85; H, 5.55; N, 11.02; O, 12.58. Found: C, 71.40; H, 5.34; N, 10.98; O, 12.28%.

*2,6-Diamino-4-phenyl-4H-thiopyran-3,5-dicarbonitrile *(**6c**). Yellow crystalline solid, Mp 192–193 °C; yield 92%, ^1^H-NMR [DMSO-d_6_], δ: ppm = 7.35 (m, 2H, Ar), 7.26 (m, 3H, Ar), 6.93 (s, 4H, 2NH_2_), 4.26 (s, 1H); ^13^C-NMR: [DMSO-d_6_], δ: ppm = 151.2 (2C), 143.5 (C), 128.7 (2C), 127.1 (C), 126.6 (2C), 118.8 (2C, CN), 71.9 (2C), 43.3 (C); MS: *m/z* % 254.1 (M+100); Anal. calcd for C_13_H_10_N_4_S (255.31): C, 61.15; H, 4.34; N, 21.94; S, 12.55. Found: C, 61.14; H, 4.02; N, 21.50; S, 12.51%.

*2-Amino-7,7-dimethyl-5-oxo-4-phenyl-5,6,7,8-tetrahydro-4H-chromene-3-carbonitrile *(**6d**). Faint yellow crystalline solid, Mp 228–230 °C; yield 90%, ^1^H-NMR [DMSO-d_6_], δ: ppm = 7.30 (m, 2H, Ar), 7.18 (m, 3H, Ar), 7.02 (s, 2H, NH_2_), 4.20 (s, 1H), 2.62 (m, 2H, CH_2_), 2.25 (m, 2H, CH_2_), 1.91 (m, 2H, CH_2_); ^13^C-NMR: [DMSO-d_6_], δ: ppm = 197.6 (C=O), 174.2 (C), 163.8 (C), 144.9 (C), 128.2 (2CH), 127.3 (2CH), 126.5 (CH), 119.4 (CN), 113.6 (C), 58.7 (C), 38.4 (CH_2_), 36.3 (C), 35.4 (CH_2_), 32.4 (C), 25.5 (2CH_3_); MS: *m/z* % 294.1 (M+100); Anal. calcd for C_18_H_18_N_2_O_2_ (294.3): C, 73.45; H, 6.16; N, 9.52; O, 10.87. Found: C, 73.67; H, 6.25; N, 9.34; O, 10.70%.

*Ethyl 6-amino-5-cyano-2,4-diphenyl-4H-pyran-3-carboxylate* (**6e**). Yellow crystalline solid, Mp 191–192 °C; yield 87%, ^1^H-NMR [DMSO-d_6_], δ 7.45 (m, 5H, Ar), 7.36 (m, 2H, Ar), 7.26 (m, 3H, Ar), 7.04 (s, 2H, NH_2_), 4.26 (s, 1H), 3.75 (q, 2H, CH_2_), 0.73 (t, 3H, CH_3_); ^13^C-NMR: [DMSO-d_6_], δ: ppm = 156.5 (C=O), 159.1 (C), 154.2 (C), 144.1 (C), 133.1 (C), 129.9 (CH), 128.6 (2CH), 128.4 (2CH), 128.0 (2CH), 127.3 (2CH), 127.1 (CH), 119.7 (CN), 108.9 (C), 60.19 (CH_2_), 56.9 (C), 40.1 (CH), 13.2 (CH_3_); MS: *m/z* % 346.1 (M+100); Anal. calcd for C_21_H_18_N_2_O_3_ (346.3): C, 72.82; H, 5.24; N, 8.09; O, 13.86. Found: C, 72.90; H, 5.28; N, 8.06; O, 13.76%.

*Benzyl 6-amino-5-cyano-2-methyl-4-phenyl-4H-pyran-3-carboxylate *(**6f**). White crystalline solid, Mp 199–200 °C; yield 65%, ^1^H-NMR [DMSO-d_6_], δ 7.26 (m, 6H, Ar), 7.13 (m, 2H, Ar), 7.08 (m, 2H, Ar), 6.95 (s, 2H, NH_2_), 5.02 (q, 2H, CH_2_), 4.34 (s, 1H), 2.34 (s, 3H, CH_3_); ^13^C-NMR: [DMSO-d_6_], δ: ppm = 156.3 (C=O), 158.3 (C), 157.4 (C), 144.8 (C), 135.7 (C), 128.5 (2CH), 128.2 (2CH), 127.8 (CH), 127.5 (2CH), 127.1 (2CH), 126.8 (CH), 119.7 (CN), 106.8 (C), 65.7 (CH_2_), 57.3 (C), 18.3 (CH_3_); MS: *m/z* % 346.2 (M+100); Anal. calcd for C_21_H_18_N_2_O_3_ (346.3): C, 72.82; H, 5.24; N, 8.09; O, 13.86. Found: C, 72.75; H, 5.13; N, 8.09; O, 14.03%.

*2-Amino-4,6-diphenyl-4H-pyran-3,5-dicarbonitrile *(**6g**). Yellow crystalline solid, Mp 162–163 °C; yield 83%, ^1^H-NMR [DMSO-d_6_], δ 7.80 (m, 2H, Ar), 7.57 (m, 3H, Ar), 7.45 (m, 2H, Ar), 7.37 (m, 2H, Ar), 7.32 (s, 2H, NH_2_), 3.44 (s, 1H); ^13^C-NMR: [DMSO-d_6_], δ: ppm = 158.5 (C), 157.6 (C), 142.2 (C), 131.7 (C), 130.0 (CH), 128.9 (2CH), 128.7 (2CH), 127.9 (CH), 127.8 (2CH), 127.7 (2CH), 118.9 (CN), 117.3 (CN), 55.6 (C); MS: *m/z* % 299.6 (M+100); Anal. calcd for C_19_H_13_N_3_O (299.3): C, 76.24; H, 4.38; N, 14.04; O, 5.35. Found: C, 76.84; H, 4.42; N, 14.01; O, 4.69%.

*2-Amino-5-oxo-4-phenyl-5,6,7,8-tetrahydro-4H-chromene-3-carbonitrile* (**6h**). Yellow crystalline solid, Mp 172–173 °C; yield 78%, ^1^H-NMR [DMSO-d_6_], δ: ppm = 7.30 (m, 2H, Ar), 7.18 (m, 3H, Ar), 7.02 (s, 2H, NH_2_), 4.20 (s, 1H), 2.62 (m, 2H, CH_2_), 2.25 (m, 2H, CH_2_), 1.91 (m, 2H, CH_2_); ^13^C-NMR: [DMSO-d_6_], δ: ppm = 198.8 (C=O), 174.4 (C), 164.4 (C), 144.8 (C), 128.3 (2CH), 127.1 (2CH), 126.5 (CH), 119.7 (CN), 113.8 (C), 58.2 (C), 36.3 (C), 35.4 (CH_2_), 26.4 (CH_2_), 19.8 (CH_2_); MS: *m/z* % 266.1 (M+100); Anal. calcd for C_16_H_14_N_2_O_2_ (266.2): C, 72.16; H, 5.30; N, 10.52; O, 12.02. Found: C, 72.04; H, 5.54; N, 10.41; O, 11.98%.

*Diethyl 5,5-dicyano-4,6-dimethyL-2-phenylcyclohexa-3,6-diene-1,3-dicarboxylate *(**7**). Pale yellow crystalline solid, Mp 210 °C; yield 25%, ^1^H-NMR [DMSO-d_6_], δ: ppm = 7.60–7.42 (m, 5H, Ar), 7, 7.02 (s, 2H, NH_2_), 3.95 (s, 1H), 3.93 (m, 4H, 2CH_2_), 2.5 (S, 3H, CH_3_), 2.31 (S, 3H, CH_3_), 1.04 (m, 6H, 2CH_3_); ^13^C-NMR: [DMSO-d_6_], δ: ppm = 167.5 (2C=O), 137 (C), 136 (C), 129 (2C), 128 (C), 127 (C), 125 (C), 123 (2C), 117 (C), 116 (C), 61 (2C), 45 (C), 30 (C), 25 (2C), 15 (2C); MS: *m/z* % 378.4 (M+100); Anal. calcd for C_22_H_22_N_2_O_4_ (378.16): C, 69.83; H, 5.86; N, 7.40; O, 16.91. Found: C, 71.2; H, 5.80; N, 7.5; O, 16.31%.

### 3.3. Experimental Procedure for the Synthesis of **4**

A mixture of benzaldehyde (**1**, 0.01 mol), malononitrile (**2**, 0.01 mol) and 10% mol L-proline, then pyrazolon-5-one (**3**, 0.01 mol) was added. The mixture was refluxed in ethanol (15 mL) for 4 h and followed by TLC. The crude compound formed was recrystallized from ethanol and further purified using column chromatography using ethyl acetate as eluent.

*6-Amino-3,4-dimethyl-4-phenyl-2,4-dihydropyrano*[2,3-c]*pyrazole-5-carbonitrile *(**4**). Yellow crystalline solid, Mp 225–226 °C; yield 81%, ^1^H-NMR [DMSO-d_6_], δ 12.1 (s, 1H, NH); 7.30 (m, 2H, Ar), 7.26 (m, 2H, Ar), 7.18 (d, *J* = 7.2 Hz, 1H, Ar), 6.78 (s, 2H, NH_2_), 1.79 (s, 3H, CH_3_), 1.76 (t, 3H, CH_3_); ^13^C-NMR: [DMSO-d_6_], δ: ppm = 159.9, 153.9, 147.3, 134.9, 128.0, 126.3, 126.0, 119.9, 116.3, 63.6, 30.1, 24.6, 13.6; MS: *m/z* % 266.1 (M+100); Anal. calcd for C_15_H_14_N_4_O (266.3): C, 67.65; H, 5.30; N, 21.04; O, 6.01. Found: C, 67.66; H, 5.01; N, 20.98, O, 6.33%.

### 3.4. Experimental Procedure for the Synthesis of **13**

A mixture of benzaldehyde (**1**, 0.01 mol), malononitrile (**2**, 0.01 mol), ethyl propiolate (**8**, 0.01 mol), pyrazolon-5-one (**3**, 0.01 mol) and L-proline (**9**, 10% mol) was added together. The mixture was refluxed in ethanol (15 mL) for 4 h, followed by TLC. The crude compound formed was recrystallized from ethanol and further purified using column chromatography using ethyl acetate as eluent.

*Ethyl-6-amino-5-cyano-4-phenyl-4H-pyran-3-carboxylate *(**13**). White crystalline solid, Mp 227–230 °C; yield 65%, ^1^H-NMR [DMSO-d_6_], δ 7.71 (s, 1H), 7.32 (m, 2H, Ar), 7.24 (m, 1H, Ar), 7.22 (m, 2H, Ar), 7.02 (s, 2H, NH_2_), 4.23 (s, 2H, NH_2_), 4.01 (m, 2H, CH_2_), 1.07 (t, 3H, CH_3_); ^13^C-NMR: [DMSO-d_6_], δ: ppm = 164.5 (CH-pyran), 164.1 (C=O), 158.6 (C), 133.3 (CH), 130.5 (2CH), 128.4 (CH), 126.5 (CH), 119.6 (CN), 11.3 (C), 61.7 (CH_2_), 57.3 (C), 30.7 (CH), 14.1 (CH_3_); MS: *m/z* % 270.1 (M+100); Anal. calcd for C_15_H_14_N_2_O_3_ (270.28): C, 66.66; H, 5.22; N, 10.36; O, 17.76. Found: C, 66.73; H, 5.34; N, 10.35, O, 17.64%.

## 4. Conclusions

L-Proline could be used as a catalyst in the reaction of active methylene ketones with α,β-unsaturated nitriles in a multicomponent reaction that leads to creation of a chiral center, and bringing about enantioselectivity for the preparation of the produced pyrans and thiopyrans in good yields.
